# Diverse nature of femtosecond laser ablation of poly(L-lactide) and the influence of filamentation on the polymer crystallization behaviour

**DOI:** 10.1038/s41598-019-39640-1

**Published:** 2019-02-28

**Authors:** Bogusz Stępak, Małgorzata Gazińska, Michał Nejbauer, Yuriy Stepanenko, Arkadiusz Antończak

**Affiliations:** 1 Laser and Fiber Electronics Group, Faculty of Electronics, Wrocław University of Science and Technology, Wybrzeże Wyspiańskiego 27, 50-370 Wrocław, Poland; 2 Department of Engineering and Technology of Polymers, Faculty of Chemistry, Wrocław University of Science and Technology, Wybrzeże Wyspiańskiego 27, 50-370 Wrocław, Poland; 3 Institute of Physical Chemistry, Polish Academy of Sciences, 01-224 Warsaw, Poland

## Abstract

Over the past few years we have witnessed growing interest in ultrafast laser micromachining of bioresorbable polymers for fabrication of medical implants and surface modification. In this paper we show that surface structuring of poly(L-lactide) with 300 fs laser pulses at 515 and 1030 nm wavelength leads to formation of defects inside the polymer as a result of laser beam filamentation. Filament-induced channels have diameter around 1 μm and length of hundreds of micrometers. SEM images of microchannels cross-sections are presented. The influence of wavelength and pulse spacing on bulk modification extent was investigated and parameters limiting filamentation were determined. We show that filamentation can be used for controlling properties of PLLA. The presence of filament-induced modifications such as empty microchannels and pressure wave-induced stress lead to increased ability of polymer to crystallize at lower temperature. Crystallization behaviour and crystal morphology after laser treatment was investigated in details using different analytical techniques such as WAXD, DSC and FTIR/ATR. Hydrolytic degradation experiment was performed. Presented method can be applied for controllable, spatially distributed modification of polymer crystallinity, crystalline phase structure and hydrolytic degradation profile.

## Introduction

The interaction of ultrashort laser pulses with band gap materials such as polymers involves nonlinear optical phenomena including multiphoton absorption, avalanche ionization or self-focusing of a beam^1^. It brings the possibility of microprocessing of aliphatic polyesters such as poly(L-lactide) (PLLA) which are frequently used in medical applications e.g. stents or scaffolds using wavelengths from infrared and visible spectral range. The other important consequence of using ultrashort pulses is significant reduction of heat affected zone due to the fact that pulse duration is shorter than the time needed for thermalisation of absorbed energy^2^. Ultrashort pulse lasers are frequently used for polymer surface structuring in bioengineering applications. There are several reports on the influence of surface topography and chemistry after laser ablation on cell response to the substrate in case of collagen/elastin blends and gelatin^3^, polyurethane/poly(lactic-co-glycolic acid)/polylacide-polyethylene glycol-polylactide (PU:PLGA:PPP) blends^4^, PLLA and polystyrene (PS)^5^, PLLA/hydroxyapatite (PLLA/HAp) composite^6^. The possibility of nanostructuring of PLLA surface by femtosecond laser-induced self-organized periodic structures was presented lately^7^. The authors focus mostly on surface chemistry and geometry as it is the most important factor of cell - substrate interaction. The influence of femtosecond laser parameters and process conditions on physicochemical properties of medical grade polylactides was also reported^8^. As it was shown, the laser wavelength used for machining of PLGA may have great impact on its biodegradability and hydrolytic degradation profile^9,10^. The investigation of laser parameters impact on different properties of polymers is very important especially in the area of biomedical applications. One of the potential possibilities of polymer modification with ultrashort pulses is generation of filaments that appear as a result of the dynamic balance between nonlinear self-focusing of a beam in dielectric media and plasma defocusing^2^. In such conditions light is propagating without diffracting in form of narrow plasma channel. In a consequence refractive index of polymer can be changed or cavity can be created along filament propagation. Such a phenomena was observed in poly(methyl methacrylate) (PMMA)^11,12^ and other polymeric media^13^. This effect is frequently presented as the way of diffractive optical elements and waveguides fabrication. Filaments generated in water film above surface or directly in material by using microscope objectives were reported as a method of micro- and nanovoid formation inside PLLA^14,15^. In the most cases filament formation is obtained by tight focusing of femtosecond laser pulses using fixed microscope objectives or by converting Gaussian to Bessel beam^16–18^. Filamentation inside PLLA was not presented till now as a way of modification of polymer crystallization behaviour and hydrolytic degradation profile.

In this study we present the possibility of very fast, comparing to setup based on fixed optics, modification of PLLA using galvanometric scanner and loosely focused Gaussian beam. This method utilizes the effect of spontaneous formation of multi filaments as a result of nonlinear self–focusing and trapping of a beam inside the polymer. We investigated here the distribution of modification within the bulk material in relation to laser pulse spatial separation and wavelength. The detailed SEM cross-sectional analysis of filamentary modifications was performed. We show the impact of modification induced by filament propagation on crystallization behaviour of biodegradable medical polymer. For this purpose we used differential scanning calorimetry (DSC), wide angle X-ray diffraction (WAXD), electron and optical microscopy and IR spectroscopy (FTIR/ATR). Moreover, we show that the bulk modification as a side-effect of surface structuring by fs laser influences the hydrolytic degradation dynamics. The laser parameters suitable for surface ablation without affecting the bulk material were also determined. Presented method has potential to be applied for fabrication of structures with gradient properties.

## Results

### Morphology of laser-induced modifications

**Figure 1:**
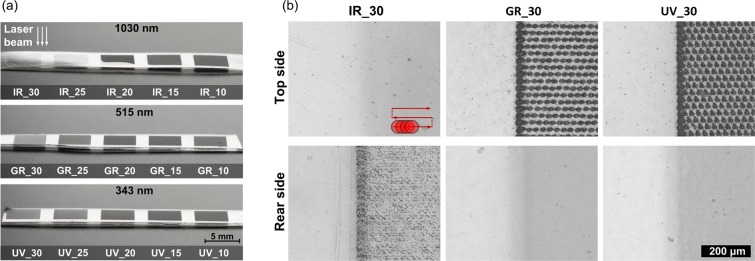
(**a**) The photographs of scanned fields showing the surface morphology for different pulse spacing at wavelength and laser fluence: 1030 nm and F = 4.4 J/cm^2^; 515 nm and F = 8.0 J/cm^2^; 343 nm and F = 4.9 J/cm^2^; (**b**) optical microscope images of the top and rear surfaces of the samples irradiated with pulse spacing 30 μm, scale bar is common for all images in Fig. 1b.

**Figure 2:**
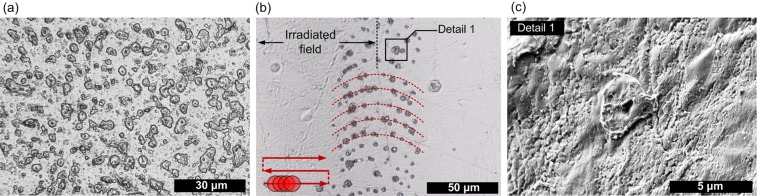
The microscopic images of: (**a**) the rear surface of sample IR_30; (**b**) the border between scanned and non-irradiated field on rear side of sample IR_10; (**c**) a single structure on rear side of sample IR_10 (SEM).

The photographs and microscopic images presented in Fig. 1 show differences in surface morphology of PLLA scanned by laser with different wavelength and overlapping of pulses where the labels of samples e.g. IR_30 indicates laser wavelength 1030 nm and distance between centres of following pulses 30 μm referred as pulse spacing. When the samples are irradiated with fluence 4.4 J/cm^2^ at 1030 nm and the pulse spacing is high (25–30 μm), top surface is not affected but modification occurs on rear side what is visible in Fig. 1b where the border between scanned and non-scanned field is presented. On the rear side of IR_30 there are multiple randomly distributed single-micron craters surrounded by material uplift (Fig. 2a). When pulse separation becomes lower and overlapping of pulses increases the top layer is being ablated and no signs of rear side modification can be observed. The boundary case between those two regimes is 20 μm pulse spacing (IR_20), where part of scanned field is ablated mainly on the top side and part modification occurs on rear side. At the beginning of the scanning procedure rear side modification is dominative however after several lines the regime switches to top side ablation (See Supplementary Fig. S1). This effect can be a result of heat accumulation and incubation of absorption centres that may enhance ablation on the top surface. When wavelength of 515 nm is applied with fluence 8.0 J/cm^2^, only top surface ablation is visible under optical microscope. Similarly at 343 nm wavelength and fluence of 4.9 J/cm^2^ top surface is effectively ablated and no rear surface modification can be observed at any pulse spacing. The top and rear sides of samples irradiated with 20 and 10 μm pulse spacing at different wavelengths are presented by analogy to Fig. 1b in Supplementary Fig. S2. The single craters obtained at 343 nm are comparable in diameter with craters obtained at 515 nm wavelength (~20 μm) (see Supplementary Fig. S3).

An interesting effect can be seen at the edge of the area scanned by 1030 nm wavelength on the rear side when pulse spacing ranges between 10 and 15 μm. The rear surface inside scanned field remains unchanged whereas at the boundary of field, structures similar to those observed in case of rear side of IR_30 sample can be observed. Those craters are aligned in circular shape and reflect beam spot shape (Fig. 2b). In Fig. 2b scanning direction is indicated. SEM image of single structure at rear side is presented in Fig. 2c.

**Figure 3:**
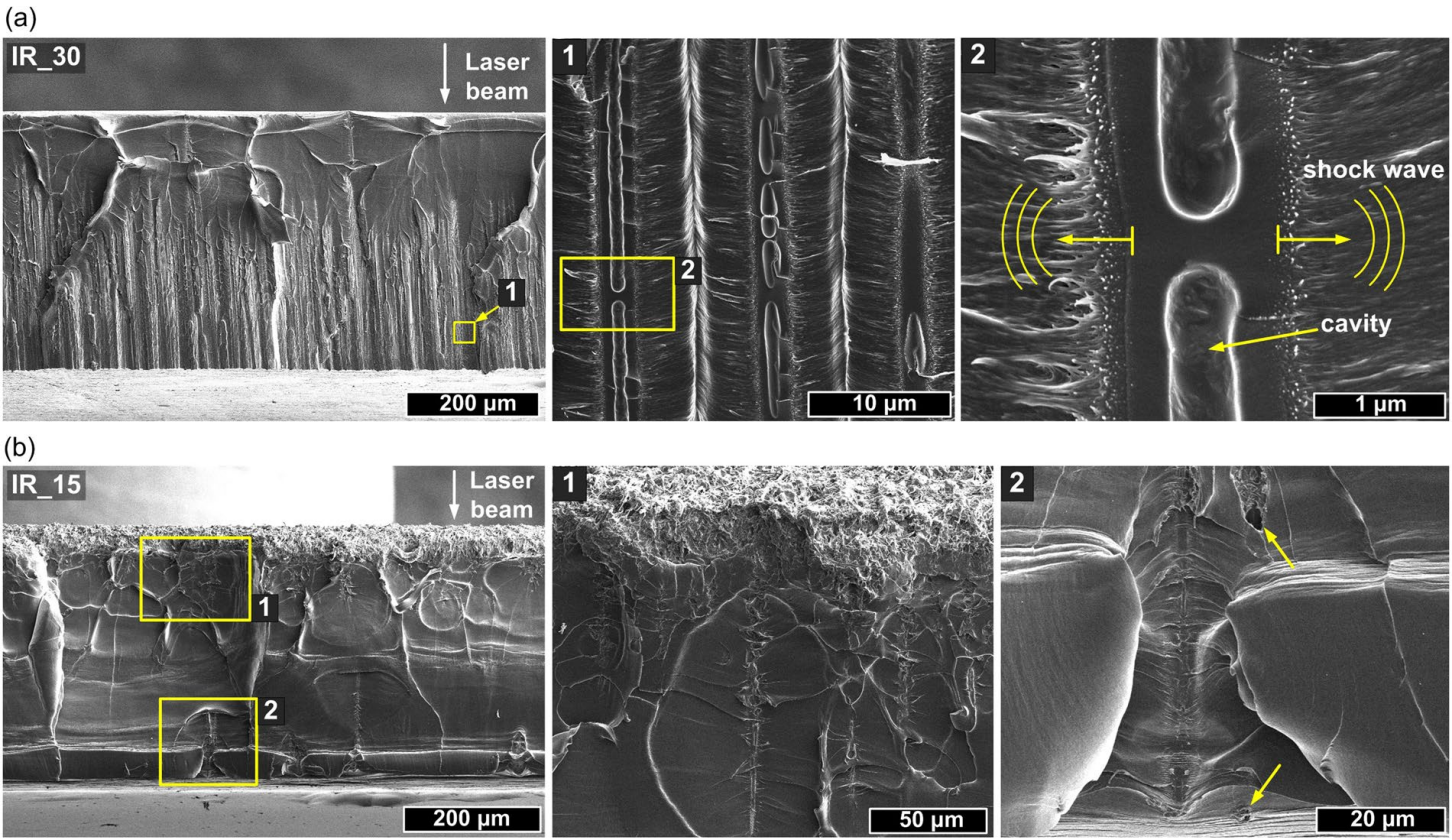
SEM images of the cross-sections of the samples modified by 1030 nm wavelength showing different microstructures created by filament propagation inside the PLLA with different pulse spacing: (**a**) IR_30 and (**b**) IR_15.

In order to investigate the cross-sections we performed mechanical braking of the samples in the middle of scanned fields. SEM images of the edge of sample IR_30 is presented in Fig. 3a. Obtained images suggest that the laser beam undergoes self-focusing inside PLLA and at certain depth starts to propagate as filaments. The modification of sample as a result of filament propagation appears ~150 μm below irradiated surface and continues down to the bottom. The average length of filaments is 270 μm. It is worth to stress here that Rayleigh range of a beam was several times higher than sample thickness and top surface was pace in focal plane. The enlarged images show that filaments cause formation of empty channel with diameter of 1.0–1.4 μm and surrounding modified zone with diameter of around 2 μm. The created voids are not continuous and in some cases resemble empty enclosed cavities (Fig. 3a). Based on microscopic images and cross-sectional analysis it can be concluded that single IR pulse causes multiple filamentary modifications which terminals are visible at rear surface.

The SEM images of IR_15 sample cross-section are presented in Fig. 3b. As can be seen higher pulse overlapping results in ablation of the top surface and formation of filaments is strongly limited. Nevertheless a few traces of filaments reaching bottom surface were found. Laser ablation with ow pulse spacing caused significant increase of surface roughness. It resulted in formation of porous structure composed of polymer fibres with diameter between 100 and 500 nm (see Supplementary Fig. S4). Interesting modifications were found also in case of sample irradiated by 515 nm wavelength.

**Figure 4:**
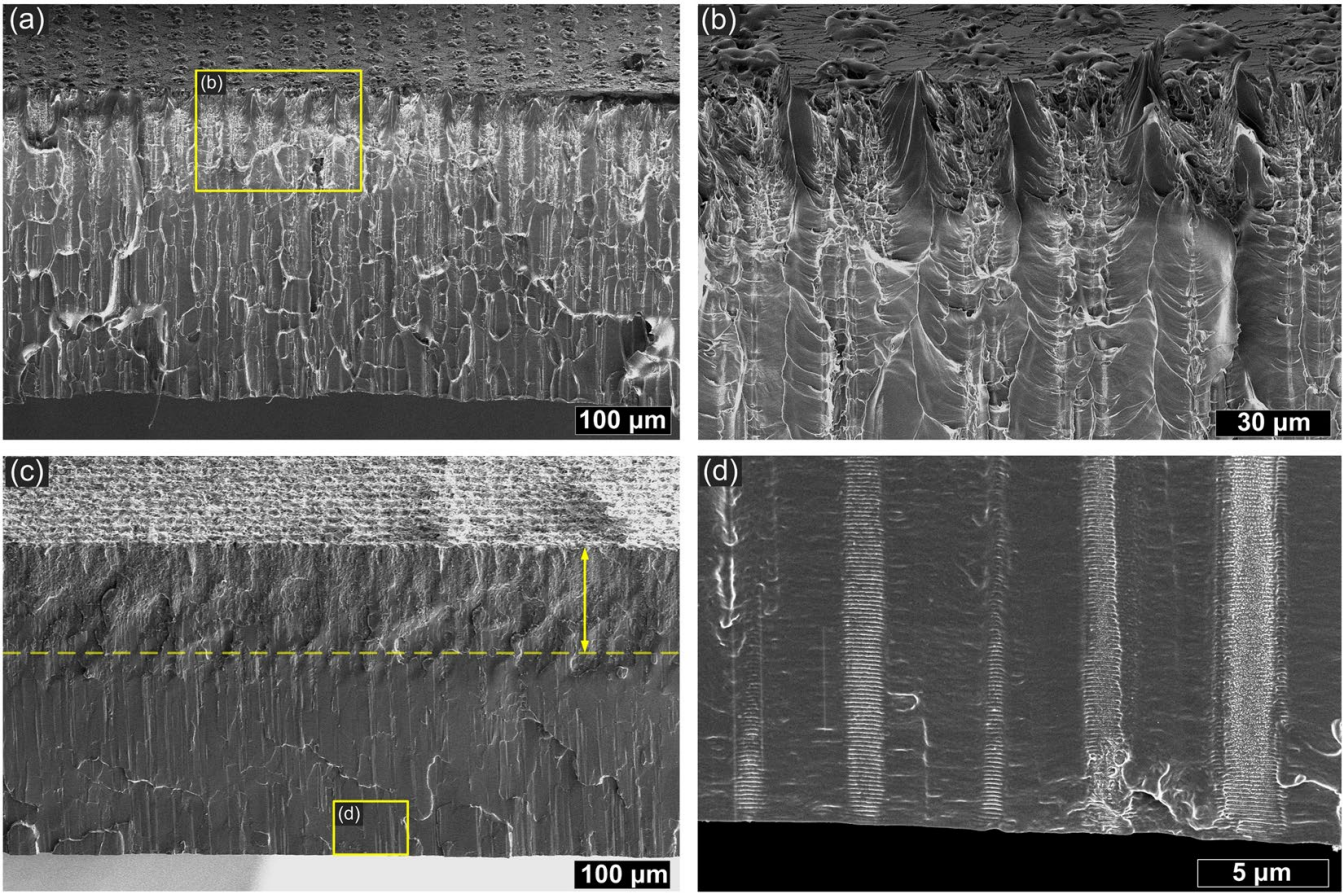
SEM images of the cross-sections of the PLLA sheet irradiated by 515 nm wavelength with different pulse spacing: (**a**) GR_30; (**b**) enlarged image; (**c**) GR_20; (**d**) enlarged image.

**Figure 5:**
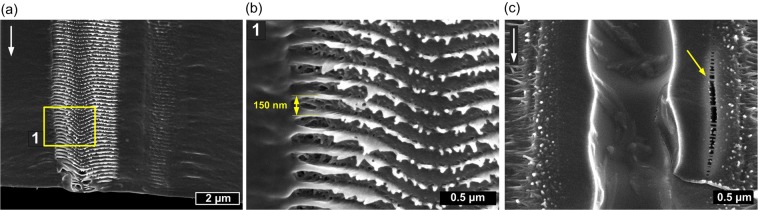
Detailed cross-sectional SEM images of selected modifications inside PLLA: (**a**) periodic nanostructures oriented transversely to the filament propagation direction (sample GR_20), the arrows indicate light propagation direction; (**b**) enlarged image of nanostructure; (**c**) microcrack in proximity of filament-induced cavity (sample IR_30).

Cross-section of the sample GR_30 (Fig. 4a) shows that filamentary modifications occur along with surface ablation. In Fig. 4b, multifilament traces are visible under the main crater. The length of filaments is comparable with PLLA sheet thickness ~420 μm. In this case it was hard however to observe well-defined capillary-like voids. When the pulse spacing is lower GR_20 filament formation is limited by analogy to 1030 nm wavelength (Fig. 4c). The strongly modified zone is however deep ~150 μm and some traces of filaments that reached sample bottom can be still observed (Fig. 4d). Cleaving of the sample GR_20 revealed periodic (Λ ≈ 150 nm) modifications oriented perpendicular to filament propagation visible in different scales in Fig. 5a,b. Such structures were observed also around filaments obtained with 1030 nm and the period of them was two times higher Λ ≈ 300 nm (see Supplementary Fig. S5). Enlarged image shows also microcracks surrounding filamentary channel caused by 1030 nm irradiation (Fig. 5c). The samples irradiated with 343 nm exhibit no traces of bulk modifications under ablated surface. Only surface ablation occurred in case of this wavelength.

### Thermal properties and crystallization behaviour of PLLA after laser treatment

During heating from glassy state above glass transition PLLA can form crystalline structure in process called cold crystallization. PLLA is polymorphic and forms different ordered structures depending on crystallization conditions. Crystallization from melt at temperatures higher than ~120 °C leads to slower formation of highly ordered α-crystals, whereas at temperature lower than ~120 °C crystals are developed much faster and are referred in the literature as α′^19^. The α′ form crystals are not perfect and are considered as conformationally disordered α-crystals with slightly increased lattice spacing. The α′-form upon further heating transforms into stable α-form. Presented results concern laser modification performed on amorphous PLLA in order to investigate its influence on cold crystallization behaviour and crystalline phase formation. Crystal form of PLLA significantly affect the application–wise properties, such as mechanical and barrier properties^20,21^. In addition, as for biodegradable polymers, biodegradability of PLLA is also influenced by the polymorphism. N. Zangg et. all demonstrated different hydrolytic degradation behaviour of α′- and α-PLLA^22^. Thus it is quite important to control the polymorphism for optimizing the properties of PLLA. Differential scanning calorimetry yields a lot of useful information about the changes introduced into material during laser irradiation as we presented previously in case of CO_2_ and excimer lasers^23–25^. Using this technique we can analyse if melting temperature (*T*_m_) of polymer changes after the process. If *T*_m_ is lower it could be a sign of racemization of polymer or decrease of molecular weight what is linked with degradation of the polymer^26^. Secondly, by the observation of the calorimetric curves in the glass transition range, we can determine among others if the material exceeded characteristic glass transition temperature (*T*_g_) during laser processing, therefore the influence of potential heat accumulation can be seen. Typically, second heating cycle is used for investigation the polymer properties, however in this case we focus mostly on first heating cycle since it yields information about changes in material thermal history and stress inside the polymer induced by laser treatment.

**Figure 6:**
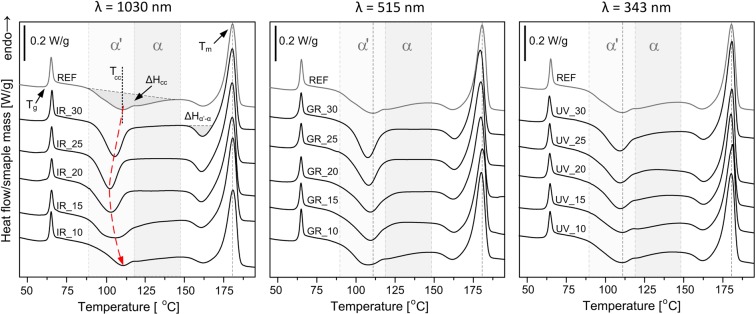
The 1^st^ heating DSC curves of PLLA modified with 1030 nm, 515 nm and 343 nm wavelengths and different pulse spacing with indicated temperature ranges for α and α′ phase crystallization.

The first heating DSC curves of unmodified PLLA and irradiated at 1030, 515 and 343 nm wavelengths with different pulse overlapping are shown in Fig. 6. The thermal parameters estimated from the first heating DSC scans are presented in Table 1. In the all presented DSC curves there are three characteristic thermal effects: glass transition with *T*_g_ at ~65 °C and following endothermic effect (enthalpy relaxation), cold crystallization in the range of 90–140 °C with peak at *T*_cc_ and melting at temperature *T*_m_ over 170 °C. Prior to the melting peak small exothermic effect is present at 160 °C. This peak indicates phase transition of crystalline α‘ phase to thermodynamically favoured α form^27^. Laser modification caused slight changes at glass transition region concerning the enthalpy relaxation peak accompanying glass transition, whereas *T*_g_ does not change significantly. The changes in melting temperature are negligibly small for the all modified samples.

**Table 1:** Parameters used for sample modification and their designation.

λ = 1030 nm, F = 4.4 J/cm^2^		λ = 515 nm, F = 8.0 J/cm^2^		λ = 343 nm, F = 4.9 J/cm^2^		Pulse Spacing (sp_x_, sp_y_) [μm]
sample designator	Overlapping (2ω_0_ = 50 μm)	sample designator	Overlapping (2ω_0_ = 25 μm)	sample designator	Overlapping (2ω_0_ = 26 μm)	
IR_30	40%	GR_30	−20%	UV_30	−15%	30
IR_25	50%	GR_25	0%	UV _25	0%	25
IR_20	60%	GR_20	20%	UV _20	23%	20
IR_15	70%	GR_15	40%	UV _15	42%	15
IR_10	80%	GR_10	60%	UV _10	62%	10

λ–Laser wavelength, F – laser fluence determined as $F=\frac{2{E}_{p}}{A}$, where *A* is beam spot area, $Ov=\frac{2{\omega }_{0}-sp}{2{\omega }_{0}}\cdot 100 \%$.

The strongest differences between unmodified and laser modified PLLA samples concern cold crystallization behaviour especially in case of 1030 nm wavelength. Cold crystallization of the reference PLLA occurs at broad temperature range with onset above glass transition at 89 °C and continue until weak exothermic effect of α′ → α transition adjacent to melting. The exotherm of cold crystallization is characterized by bimodal peak with distinguishable two maxima at 109.9 °C and 122.0 °C. It is known that during cold crystallization at lower temperatures (at high supercooling) α′ phase crystallizes^28^. For 1030 nm wavelength modified PLLA with pulse separation 30–15 μm cold crystallization exotherms have a single, narrow peak at lower temperature. Moreover, disappearance of high temperature contribution of cold crystallization is accompanied by increase of the enthalpy of α′ → α transition (ΔH_α′-α_). This also indicates that more α′ phase is formed during cold crystallization of laser modified sample at 1030 nm. The enthalpy of cold crystallization (ΔH_cc_) after laser modification (for IR_30 – IR_15) is lower than for the unmodified PLLA, because of lack of high temperature contribution of crystallization transition. The lowest peak temperature of cold crystallization (102.9 °C) was found for the sample with 20 μm pulse spacing. There is general tendency for 1030 nm wavelength that along with the increase of laser pulse overlapping the changes in DSC curves between reference PLLA and laser treated samples are firstly intensifying for pulse spacing 30 and 25 μm and in a second stage they are gradually decreasing with pulse overlapping increase. This is connected with density of filamentary modifications. The narrower cold crystallization peak with maximum at lower temperature is observed for all samples irradiated by 1030 nm wavelength excluding IR_10 which reveals similar cold crystallization behaviour and the values of ΔH_cc_ are similar to the reference PLLA (see Supplementary Table S1).

Cold crystallization exotherms of PLLA modified with 515 nm are similar to those modified with 1030 nm wavelength. Peak temperature of cold crystallization is also moved to lower temperatures than the reference but with lower extent. For 515 nm, the changes in cold crystallization region are the most intensive for 30 μm spacing and then gradually decrease with pulse overlapping increase. The DSC curve of GR_10 sample resembles reference PLLA by analogy to IR_10. For PLLA samples modified with 343 nm wavelength changes at cold crystallization range the weakest. The high temperature contribution of cold crystallization is present for all UV ablated samples. In general DSC curves of modified samples are similar to the reference PLLA. The changes are visible mainly in case of UV_30 and UV_25 for which high temperature cold crystallization contribution is lower and ΔH_α′-α_ enthalpy is slightly higher (see Supplementary Table S1).

In the second heating cycle DSC curves of laser treated samples resemble reference sample with bimodal cold crystallization peak (see Supplementary Fig. S6). Laser modification does not cause any permanent change in melting and glass transition region that could be visible in second heating cycle. The thermal parameters obtained based on cooling cycle and second heating cycle are presented in Supplementary Tables S2 and S3.

### Crystalline structure of PLLA developed after IR femtosecond pulse irradiation

**Figure 7:**
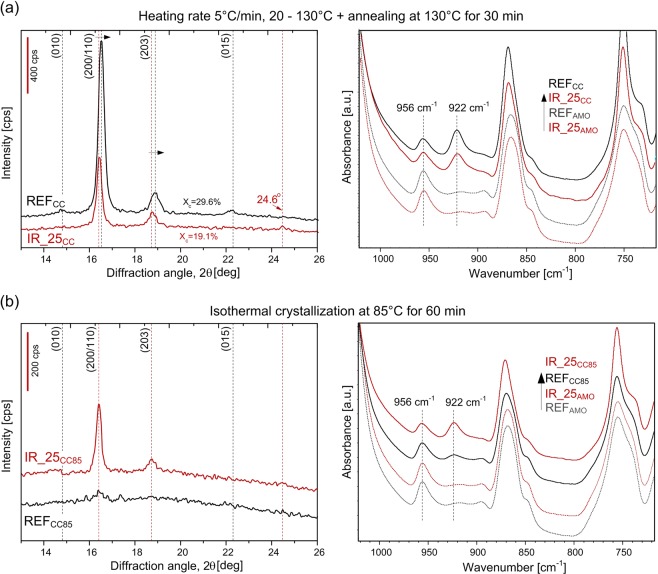
WAXD patterns and FTIR/ATR spectra of IR_25 and REF sample after subsequent conditioning: (**a**) nonisothermal (IR_25_CC_) and (**b**) isothermal cold crystallization (IR_25_CC85_).

To investigate in detail the crystal structure developed during subsequent heating of samples modified by filament propagation we performed WAXD and FTIR in ATR. Based on DSC results the reference PLLA (REF) and IR_25 samples were selected for experiments and then conditioned according to two different procedures. In the first procedure that resembles DSC heating scan samples were nonisothermally crystallized by heating with the rate of 5 °C/min from room temperature to 130 °C and annealed afterwards at 130 °C for 30 min. Samples after nonisothermal cold crystallization are abbreviated as REF_CC_ and IR_25_CC_. According to second procedure samples were crystallized isothermally at 85 °C for 60 min. The samples after isothermal crystallization are abbreviated as REF_CC85_ and IR_25_CC85_. For clarity the samples before conditioning were renamed as REF_AMO_ and IR_25_AMO_ because they are mostly amorphous. Indexing of the observed reflections is based on the crystal structure reported for the α form^29^. Figure 7a presents WAXD patterns and FITR/ATR spectra of the sample REF_AMO_ and IR_25_AMO_ after subsequent nonisothermal conditioning. Notable differences in WAXD patterns are as follows:

1. integral intensity of the reference PLLA is higher than for laser modified IR_25_CC_ (crystallinity X_c_ of REF_CC_ and IR_25_CC_ are 29.6% and 19.1%, respectively);
2. for IR_25_cc_ angular position of the two strong reflections of (200/110) and (203) are shifted to lower 2θ and widths of this reflections are narrower;
3. for the REF_cc_ some weak diffraction peaks at 14.8°, 22.2°, 28.9° indicative of (010), (015), (018) reflections characteristic of stable α crystals are visible (see also Supplementary Fig. S7)^29,30^.

The angular position of (200/110) and (203) and the presence of the reflection at 24.6° indicate that mostly α′- form crystal was developed during cold crystallization of laser modified PLLA IR_25_cc_. In case of REF_cc_ the profiles of (200/110) and (203) reflections can be resolved into two components originating from the α′ and α phases (see Supplementary Fig. S7). These findings agree with DSC results and confirm that laser modified PLLA under subsequent nonisothermal heating crystallize in α′ form whereas for the reference PLLA mixture of α and α′ forms with higher overall crystallinity is developed.

In the ATR spectra the changes were observed mostly in the spectral region related to polymer crystallinity. Both reference REF_AMO_ and laser treated sample IR_25_AMO_ which were not conditioned exhibits intense 956 cm^−1^ band representing amorphous phase (Fig. 7a). The intensity of the band representing α - crystals at 922 cm^−1^^31^ is higher in case of samples after conditioning. It can be seen that crystallinity of reference sample REF_cc_ is higher than the sample IR_25_CC_ what is in line with WAXD results. Additionally the difference in crystalline phase morphology was analysed by deconvolution of C=O stretching band at 1750 cm^−1^ which can be split into four subbands due to the helical conformation of the chain (see Supplementary Fig. S8). The ratio between those components can yield information about crystallinity as well as crystalline phase perfection^32^. The result of deconvolution also indicates higher order of crystalline phase and therefore higher amount of α crystals in case of REF_cc_.

In contrast, when conditioning process is performed isothermally at lower temperature of 85 °C for 60 min the final result is opposite. Based on WAXD patterns (Fig. 7b) it can be concluded that reference PLLA remained amorphous (REF_CC85_), whereas for IR_25_CC85_ crystalline reflections at 16.4° and 18.7° are visible and its angular position indicates α′ crystal structure formation. Higher crystallinity of the laser modified sample IR_25_CC85_ after isothermal heating is clearly visible also in FTIR spectra (Fig. 7b).

### Hydrolytic degradation rate

**Figure 8:**
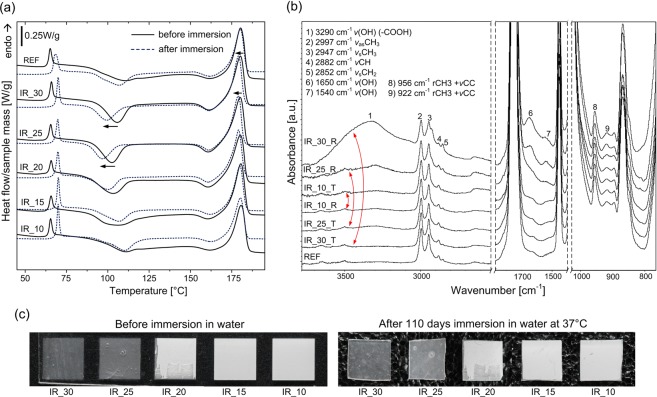
(**a**) DSC curves of the first heating cycle before and (dotted line) after water immersion; (**b**) FTIR/ATR spectra of selected samples after hydrolysis measured on both rear (R) and top (T) side; (**c**) photographs of the same samples before and after hydrolysis.

As previously reported, femtosecond laser ablation of PLGA caused increased degradation rate and notable differences between reference and modified sample only after 8 days^10^. Because used PLLA has much longer degradation period (~3 years *vs* few weeks) we have run the experiment for 110 days in order to have first insight in the impact of 1030 nm wavelength induced filamentary modifications inside the material on the hydrolytic degradation rate. As shown in SEM images, longitudinal modifications obtained with 1030 nm wavelength are frequently empty voids that can be penetrated by water. Moreover, the material at the walls of filament-induced channel is supposed to be modified in the highest extent. Therefore higher degradation rate or faster crystallization in water environment could be expected. After over three months of immersion the samples were dried and the weight loss was measured. We noted no weight loss with accuracy of 0.1 mg. The weight of the samples was in the range of 10–11 mg. DSC analysis of immersed samples did not reveal any significant changes in melting temperature *T*_m_, however, increase of *T*_g_ was observed in case of the all samples including reference PLLA (Fig. 8a). The enthalpy relaxation is higher for all incubated samples what indicates short range ordering during hydrolysis in 37 °C environment temperature. The differences in *T*_cc_ are stronger in case of samples rich of filamentary modifications (IR_30, IR_25, IR_20). The tendency in DSC curve shapes caused by different pulse separation is analogical for the samples before and after water immersion. Detailed thermal parameters based on first heating cycle are presented in Supplementary Table S4 available.

The appearance of the samples surface and geometry also did not change significantly after hydrolytic degradation experiment (Fig. 8c). The microscopic observations, weight loss measurements and DSC curves suggest weak influence of laser irradiation on hydrolytic degradation rate. However, by applying surface sensitive FITR/ATR analysis we observed notable differences between spectra of the samples IR_30, IR_25 measured on rear and top side. As presented in Fig. 8b, the rear sides of the samples with high number of empty filamentary channels are rich of hydroxyl groups (-OH) bounded with –COOH^33^. The spectra of the top sides of the same samples are very similar to reference (nonirradiated) sample and the rear side of sample IR_10. Increased number of hydroxyl groups was found also on the top side of effectively ablated samples IR_10, IR_15, however the changes are much weaker than in case of filament-induced microchannels. Together with wide absorption band centered at 3290 cm^−1^, we observed increase of intensity of bands at 1650 cm^−1^ and 1540 cm^−1^ (Fig. 8b). According to literature, those bands can be linked to adsorbed H_2_O molecules and unresolved hydroxyl groups^34–36^. This is the sign of higher water uptake. The ratio between intensity of bands at 922 and 956 cm^−1^ is similar for all the samples what indicates no significant influence of laser modification on PLLA crystallinity developed during immersion in water.

## Discussion

The microscopic observations revealed the strong influence of laser wavelength and pulse overlapping on the extent and nature of modifications inside PLLA. In the domain of ultrashort pulses, absorption coefficient of band gap material is proportional to *I*^K−1^, where *I* is intensity and *K* is the number of photons required to exceed energy bandgap which in case of PLLA is equal to ~5 eV. When the photon energy is higher, the increase of the free carrier population required for ablation is faster and more localized. Ionized material acts as shielding and limits the light penetration into the sample what can be observed when 343 nm wavelength is used. We observed deep light penetration of samples at 515 and 1030 nm wavelengths in form of filaments. As the dynamics of self-focusing of a beam depends on initial beam Rayleigh range, wavelength, nonlinear and linear refractive index of medium and pulse peak power, we observe different lengths and starting points of filament propagation at 1030 and 515 nm. The used setup was not optimized for filament generation, hence multifilament appearance is a result of beam self-organization inside the material. When the specific for the material critical power P_cr_ is exceeded, the beam may break into several filaments each carrying the power comparable with critical power^37^. The effect of a breakup of cylindrical symmetry is connected with weak or random inhomogeneity in the input laser pulse^2^. Single infrared 43 μJ pulse deposition resulted in more than dozen of filamentary modifications. We observed filaments in amorphous samples, however, in other investigation where high crystallinity PLLA/HAp composite was irradiated by 515 nm femtosecond pulses such modifications did not occur^6^. Supramolecular structure of crystalline domain that cause strong scattering of light limits the filament formation. We observed terminals of filamentary channels at rear side of samples. The uplift of the material at the end of the channels (Fig. 2c) is probably a result of material ejection. The SEM images of sample cross-section showed multiple cavities with random distribution along single filament. Those observations suggest melting of the material. Recently, the investigation on filaments induced by tight focusing femtosecond laser beam inside PMMA led to conclusion that thermal effects that enhance fluidity of polymer play important role in formation of a void^38^. The propagation of shock wave as result of sudden thermalisation of energy carried by electrons along filament inside PMMA has been observed in pump-probe experiment where shock wave propagation was observed to propagate both cylindrically around the filament and toward the sample surface^16^. Microscopic observation revealed that at high overlapping of pulses, the ablated surface which is being created during the process causes distortion of wavefront and limits the filament formation. When pulses are deposited close to each other the incubation of defects in material leads to stronger absorption. At the border of field scanned at 1030 nm we observed some terminals of filaments aligned in a circular shape (Fig. 2b). This effect is a result of lower overlapping at the boundary what allows for enhanced light penetration. Circular shape may also result from the refractive index change caused by previous pulses.

Interesting phenomenon was observed in case of 515 nm wavelength where both surface ablation and filament formation underneath is present (Fig. 4b). This was possible because macroscopic effect of 300 fs pulse deposition occurs after the energy thermalisation >1 ps. Therefore the crater that would distort wavefront appears after the pulse. At first stage of laser-material interaction, the free carrier population is not high enough to block light penetration. Filaments appear close to the surface suggesting stronger self-focusing at 515 nm than at 1030 nm. We suppose that first part of pulse penetrates the material deeply and propagates as filament while the second part of the pulse causes ablation of surface. We observed also an effect of perpendicular to filament ripple formation (Fig. 5 and Supplementary Fig. S5). As these regular nanodefects correspond to laser wavelength they can be a result of periodic changes of electron density. Those instabilities resembles characteristic for ultrashort pulse high spatial frequency laser laser-induced periodic surface structures (LIPSS) which commonly occurs at the surface^7,39^ but were also observed inside dielectrics^40^.

The DSC analysis proved no bulk modification of the samples irradiated with high overlapping at 1030 nm and 515 nm which is in line with microscopic observations. Calorimetric curves confirm that at high pulse overlapping a kind of shield zone that protects the material inside is being formed. The material at the ablated surface does not influence the DSC curves in significant way. When 343 nm wavelength is used, the bulk modifications are negligibly small comparing to 515 and 1030 nm wavelengths. It confirms no filament formation and no other significant bulk modifications. As DSC revealed the strongest increase of cold crystallization rate at low temperature was obtained for samples IR_25 and GR_30. For those samples the number and the length of filaments were the highest. A fast development of α′ crystalline phase at low temperature in case of laser irradiated samples inhibits the crystallization of α phase over 115 °C during first heating cycle. Only after exceeding certain temperature of ~155 °C further ordering is possible. It shows that the laser modification can selectively tune crystallization rate as well as crystalline structure in spatial sense. The samples with empty channels are brittle, however, after subsequent crystallization, their mechanical properties are recovered. The mechanical properties should be however investigated in a framework of further research. The molecular weight is one of the key variables governing crystallization kinetics of PLLA. With decrease of molecular weight the cold crystallization peak shifts to lower temperature, owing to the increase of crystallization rate^41^. Modification with 1030, 515 and 343 nm, however did not influence melting temperature (*T*_m_) during neither first nor second DSC heating cycle. Moreover, in the second heating cycle the all DSC curves resemble unmodified material (see Supplementary Fig. S6). Inside filament-induced channels there are possibly low molecular weight decomposition products however the amount of this fraction is too small to detect it using DSC. The change in cold crystallization behaviour is therefore not connected with molecular weight decrease within entire sample but presence of the filament-induced empty cavities. Nucleating effect of filaments can be explained by three possible ways. Inside the filaments there is possibly a small fraction of monomers or oligomers from laser-induced degradation of PLLA and they cause increase of cold crystallization rate. Second possible explanation is related to presence of new surfaces introduced into bulk material. Nucleation mechanism in bulk is different than surface nucleation from micro or nano pores^42^. Finally, the compaction of the material around filaments as a result of cylindrical shock wave propagation may also affect the crystallization dynamics.

The ATR measurements of the samples directly after laser treatment, before water immersion, on the top and rear sides showed no explicit signs of degradation of the material in form of new IR absorption bands at 811, 990, 1410 cm^−1^ (-CH=CH_2_) that were observed e.g. in case of CO_2_ laser modification^24^. The differences between ATR spectra of samples modified by 1030 nm are visible after hydrolytic degradation experiment. The much higher content of -COOH groups and water molecules at surface with filamentary channel terminations suggest that either the material ejected is more susceptible to hydrolytic attack or the geometry of the sample facilitates water uptake into the microcavities or both those factors enhances the modification of the rear surface of samples IR_30 and IR_25 in water environment. The sample IR_10 for which the direct ablation caused strong modification of top surface geometry (see Supplementary Fig. S4) revealed even weaker susceptibility to hydrolytic attack which is a surprising result. Regarding the changes caused by hydrolysis within entire samples, DSC revealed stronger shifts of cold crystallization temperature T_cc_ and T_m_ in case of samples IR_30 and IR_25. These changes suggest the possible decrease of molecular weight in water environment enhanced by filament – induced microchannels. The results of hydrolytic degradation experiment suggest that microchannels formed by filament propagation accelerate hydrolysis stronger than multipulse surface ablation. Laser – induced cavities influence strongly the crystallization behaviour and the may be used to obtain certain crystal structure perfection in spatially selected regions. The ordering of crystalline phase in turn may influence even more the hydrolytic degradation profile. As it was reported, overall crystallinity as well as crystal form of PLLA has an impact on hydrolytic degradation profile while the crystal forms α or α′ governs the degradation profile at the end of process^22^. We believe that by using method presented here, adjusting filament density and applying proper conditioning, gradient properties of PLLA can be achieved in terms of both crystallinity and hydrolytic degradation profile. This aspect however requires further investigations.

## Summary

The femtosecond laser-induced bulk modifications inside PLLA were characterized. As microscopic analysis revealed, using constant pulse duration of 300 fs it is possible to control the modification extent of PLLA and its spatial distribution by changing the laser wavelength and overlapping of pulses. Calorimetric curves showed that the intensity of bulk modification in sense of cold crystallization behaviour can be adjusted by overlapping of pulses. The conclusions from microscopic cross-sectional analysis are in good agreement with DSC results. The filamentation inside the material can be limited by appropriate pulse overlapping when the laser process is aimed at only surface modification. Using UV wavelength, only surface modification was obtained. We presented detailed analysis of crystalline phase developed during subsequent heating after laser modification. Filamentary modifications act as crystallization nuclei and cause enhanced polymer crystallization at lower temperature and formation mostly α′ crystal structures. In consequence, subsequent conditioning can induce different crystalline phase morphology in different locations of specimen hence induce gradient mechanical and thermal properties. This method can be applied for controllable change of polymer crystallinity and hydrolytic degradation profile with additional thermal post processing after laser treatment. Furthermore, we observed that filamentary modifications accelerate hydrolysis stronger than multipulse surface ablation. For filament assisted bulk modification of PLLA crystallization behaviour the best solution was 1030 nm wavelength as it does not cause surface ablation and promotes formation of multiple empty microcavities from a single laser pulse.

## Methods

### Materials

We used poly(L-lactide) RESOMER® L210 S from Evonik. The material was processed in hydraulic press in order to achieve amorphous polymer sheets. Before pressing the polymer was dried for 3 h at 100 °C to reduce its moisture. The processing temperature was 190 °C and pressure ~50 bar. The material was cooled down inside the press at a rate of 20 °C/min. The thickness of the samples was 420 μm. The molecular weight of the polymer sheet was determined in previous studies where the samples were prepared according to the same procedure: M_n_ = 247 500 g/mol, M_w_ = 493 400 g/mol, PDI = 2.0^23,24^. The values are expressed according to polystyrene standards.

### Laser modification

**Figure 9:**
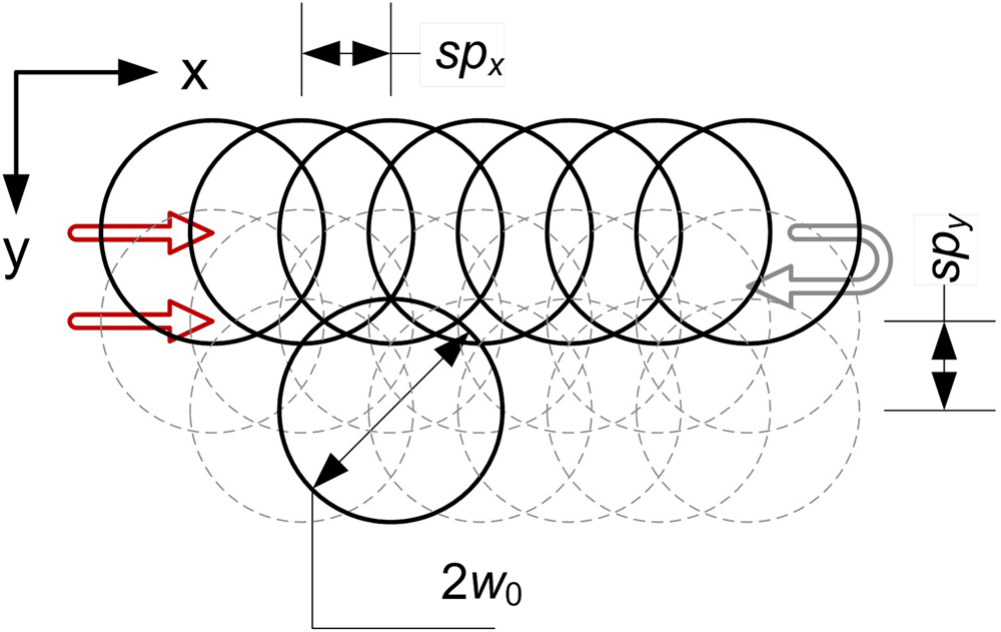
Scanning procedure where *sp*_*x*_ = *sp*_*y*_ is pulse spacing and *2ω*_0_ is laser spot diameter.

In the investigation we used a femtosecond laser source based on chirped pulse amplification with pulse duration of ~300 fs after passing focusing optics. The femtosecond pulses generated by an oscillator based on all-PM-fibre laser mode-locked with a nonlinear loop mirror^43^ were amplified in a two-stage amplifier using an ytterbium doped photonics crystal fibre (rod) in the final booster stage. The system was developed in the Institute of Physical Chemistry Polish Academy of Sciences. The maximum average power available at the fundamental wavelength was 30 W. We used constant pulse energy of 43.3 μJ at 1030 nm, 20.7 μJ at 515 nm and 13.0 μJ at 343 nm. The pulse repetition frequency was fixed at 10 kHz. Samples were mounted in such a way to avoid influence of substrate ablation underneath or light reflection. Processing was performed in ambient air. The beam was delivered to the workpiece through a galvanometric scanner and focusing optics with the focal length of f = 163 mm at 1030 nm, 515 nm and f = 250 mm at 343 nm. The resulting beam waist was 50 μm, 25 μm and 26 μm. The polarization was linear. The varying parameter was pulse overlapping which was changed in both scanning directions. Different overlapping of pulses we obtained by changing pulse spatial separation *sp*_x_ and *sp*_y_ (Fig. 9). Spatial separation of pulses was changed between 10 and 30 μm with 5 μm step. Resulting pulse overlapping depending on beam spot size is presented in Table 1. Overlapping factor was calculated as the percentage of the overlapped diameter in relation to total beam diameter in scanning direction.

### Differential scanning calorimetry

Differential scanning calorimetry (DSC) measurements were performed using the Mettler Toledo DSC1 system, coupled with Huber TC 100 intracooler. The instrument was calibrated using indium (T_m_ = 156.6 °C, ΔH_m_ = 28.45 J/g) and zinc (T_m_ = 419.7 °C, ΔH_m_ = 107.00 J/g) standards. Samples (~3.5 mg) were measured in the 40 μL aluminium pans under a constant nitrogen purge (60 mL/min) from 0 °C to 200 °C. After the heating cycle, the samples were thermally equilibrated at 200 °C for 5 minutes and cooled down to 0 °C. Second heating scan was also performed. Both heating and cooling rates were set to 5 °C/min. The glass transition temperature (T_g_) from heating and cooling scans were taken as the inflection point in the heat flux curve. Experimental data was processed using the generic STAR^e^ computer program. For the purpose of data presentation, the DSC profiles were exported to OriginPro 64 (v. 9.0) as ASCII files.

### WADX analysis

WAXD (wide angle X-ray diffraction) experiments were done at room temperature on Rigaku Ultima IV diffractometer (Bragg – Brentano geometry) with Ni filtered Cu_Kα_ (λ = 1.54178 Å) radiation generated by sealed X-ray tube. The radiation source was powered by a generator operated at 40 kV and 30 mA. Data were collected within the range of 2θ from 1.5° to 65.0° in a fixed time-scan mode with counting time 5.0 s and step width 0.05°. The background corrected WAXD patterns were resolved into Lorenzian shape diffraction peaks and diffusion maxima by using the Levenber-Marquardt non-linear fitting procedure implemented on OriginPro 9.0 software. The degree of crystallinity (X_c_) was calculated according to the following relation:

1 $${X}_{c}=\frac{{\sum }^{}{A}_{c}}{{\sum }^{}{A}_{c}+{\sum }^{}{A}_{a}}\times 100 \% ,$$

where A_c_ and A_a_ represent the integrated intensities under the crystalline reflections and the integrated intensities under diffuse maxima.

### IR spectroscopy

The ATR-FTIR spectra were recorded using the FTIR Nicolet TM 8700 spectrometer with the Smart Orbit Diamond ATR accessory (Thermo Fisher Scientific Inc.) within the range of 700 ÷ 4000 cm^−1^ and with a 0.48 cm^−1^ step. The reference and laser treated samples were measured before and after crystallization in different conditions. Each sample was measured on both sides. A fitting procedure was performed using Omnic 8.3.103 software.

### Hydrolytic degradation experiment

The samples in form of laser scanned fields were cut out from polymer sheet and the mass of them was determined using high resolution laboratory balance (accuracy 0.1 mg). Each sample was measured 5 times. Then the samples were placed in separate glass probes filled with distilled water. Samples were stored for 110 day in laboratory furnace at 37 °C in closed probes. Each day the probes with samples were placed in a laboratory shaker for 15 min. After testes, samples were dried in exsiccator under 100 mbar pressure for 8 hours and additional in furnace in 40 °C for 8 hours. After drying, samples were analysed using laboratory balance in order to measure mass loss. The dried samples were also analysed by means of DSC and FTIR/ATR.

## Supplementary information

Supplementary Information
