# Supplementary material for: Diverse nature of femtosecond laser ablation of poly(L-lactide) and the influence of filamentation on the polymer crystallization behaviour
Source: Sci Rep. 2019 Feb 28;9:3069. doi: 10.1038/s41598-019-39640-1 (PMC6395729; doi:10.1038/s41598-019-39640-1)
Supplement: Supplementary file 1 — Supplementary Information [file 41598_2019_39640_MOESM1_ESM.pdf]

Supplementary Information

Diverse nature of femtosecond laser ablation of poly(L-lactide) and the influence of filamentation on the polymer crystallization behaviour

Bogusz Stępak[1], Małgorzata Gazińska[2], Michał Nejbauer[3], Yuriy Stepanenko[3], Arkadiusz Antończak[1]*

[1]Laser and Fibre Electronics Group, Faculty of Electronics, [2]Department of Engineering and Technology of Polymers ,Faculty of Chemistry, Wrocław University of Science and Technology, Wyb. Wyspiańskiego 27, 50-370 Wrocław, Poland, [3]Institute of Physical Chemistry, Polish Academy of Sciences, 01-224 Warsaw, Poland
*bogusz.stepak@pwr.edu.pl

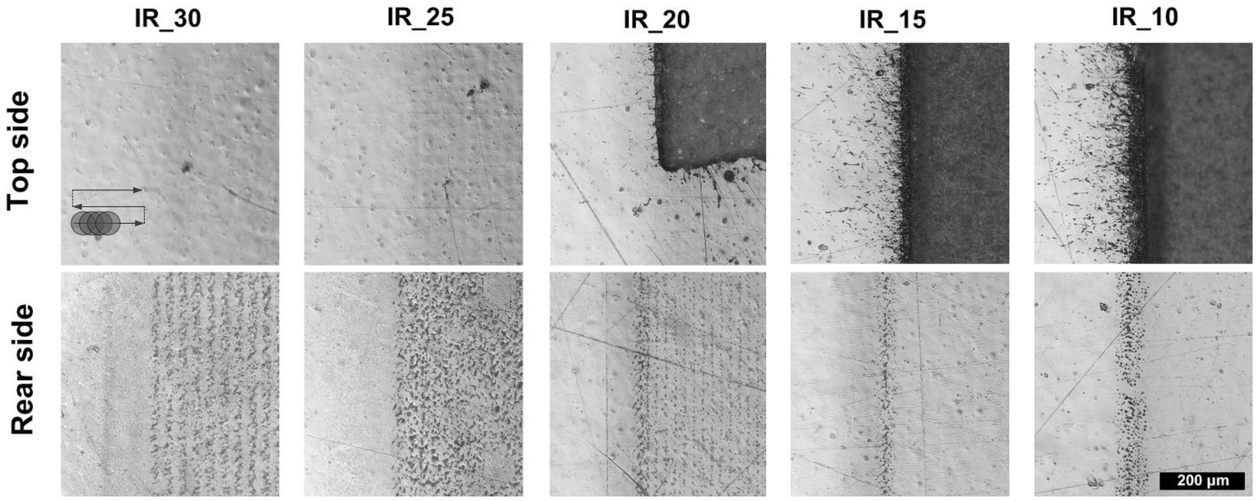

Supplementary Figure S1. Evolution of rear and top surface modification of the samples irradiated by 1030 nm wavelength. Scale bar is common for presented microscopic images.

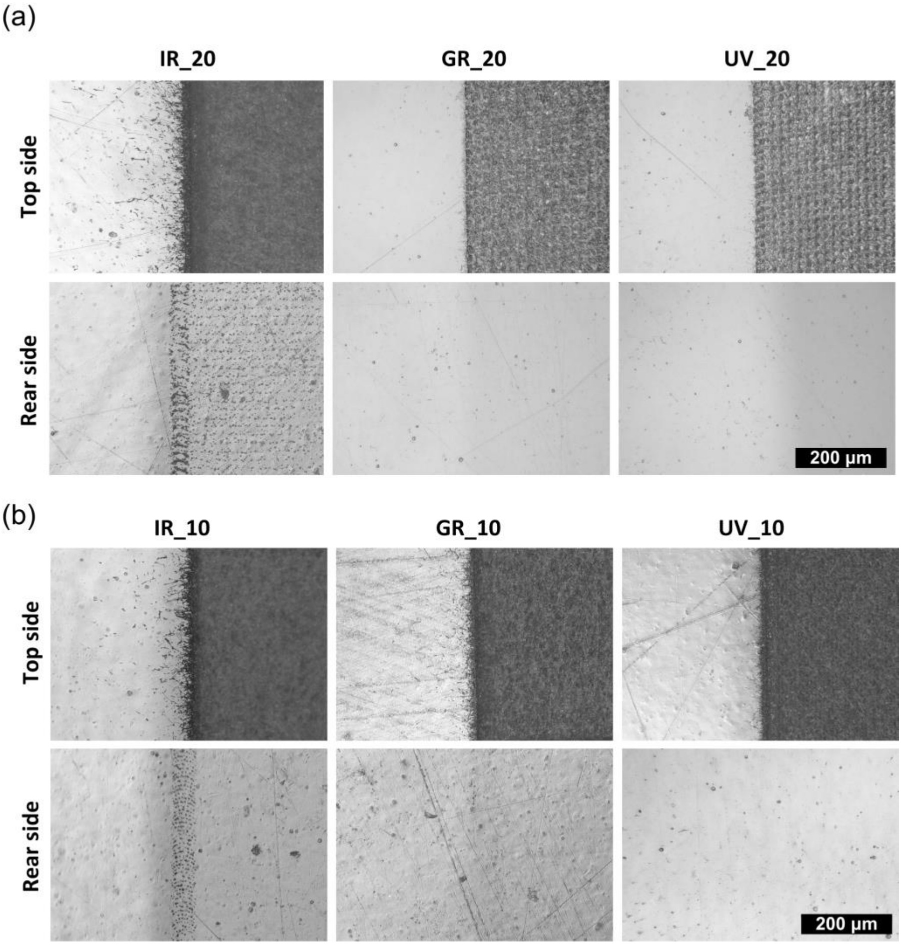

Supplementary Figure S2. Optical microscope images of the top and rear surfaces of the samples irradiated at three wavelengths with pulse spacing a) 20 µm and b) 10 µm.

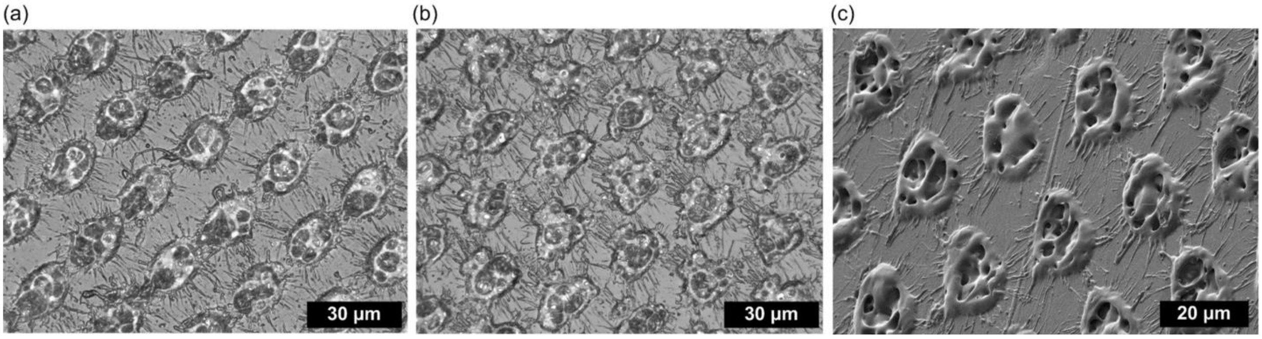

Supplementary Figure S3. The craters at the top surface of sample a) GR_30 and b) UV_30, c) SEM image of craters at the surface of UV_30 sample

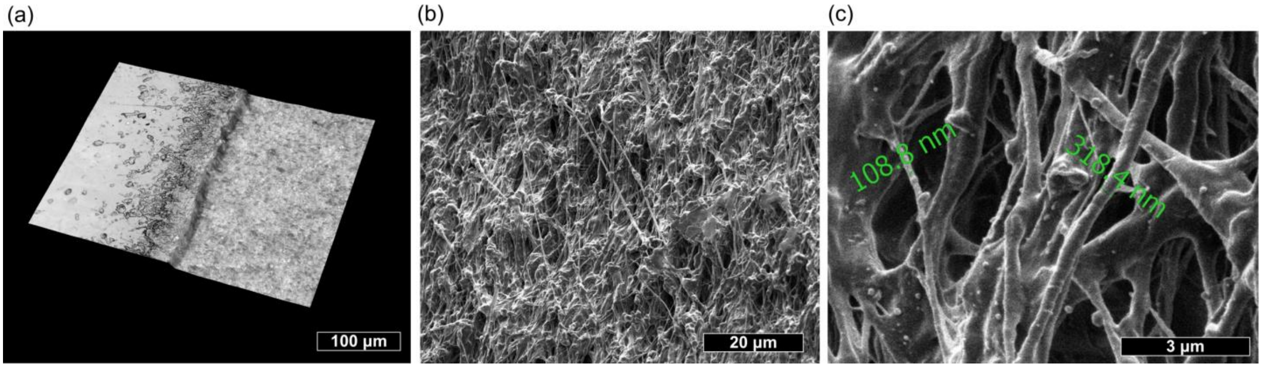

Supplementary Figure S4. The images of top ablated surface at 1030 nm wavelength and with low pulse spacing: a) optical microscope 3D image of the edge of ablated field, b) SEM image of the ablated surface, c) enlarged nanofiber formed during ablation of top surface

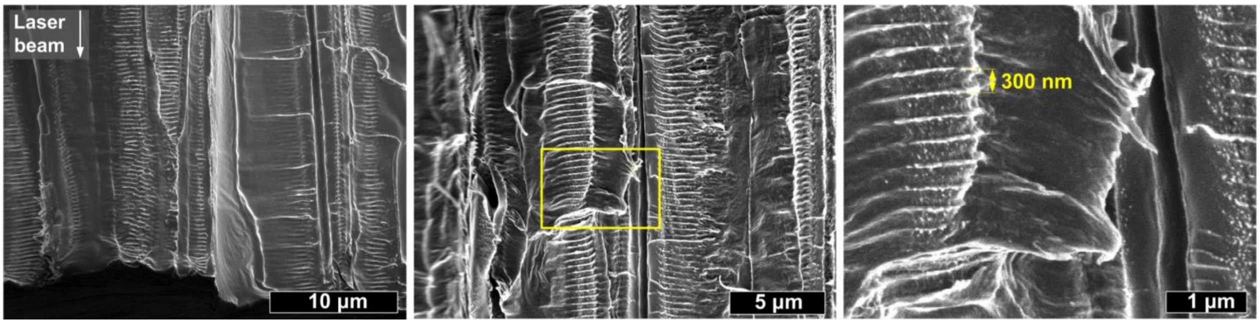

Supplementary Figure S5. Detailed cross-sectional SEM images of periodic nanostructures formed as a result of filament propagation inside PLLA induced by laser pulse at 1030 nm (sample IR_30) presented in different scales

Supplementary Table S1. The thermal parameters of laser modified PLLA obtained based on the first heating DSC scan

sample	T_g [^oC]	T_{cc} [^oC]	ΔH_{cc} [J/g]	$T_{\alpha'-\alpha}$ [^oC]	$\Delta H_{\alpha'-\alpha}$ [J/g]	T_m [^oC]	ΔH_m [J/g]
REF	64.4	109.9	**-36.6**	161.2	**-2.6**	180.7	41.7
IR_30	65.0	105.5	**-25.3**	161.4	**-5.7**	180.6	42.3
IR_25	64.7	102.5	**-28.2**	160.7	**-6.3**	180.4	44.4
IR_20	64.7	102.9	**-21.0**	160.5	**-5.1**	180.5	44.8
IR_15	64.6	105.3	**-21.2**	160.6	**-4.0**	180.7	44.3
IR_10	64.5	110.5	**-33.5**	161.3	**-1.6**	180.7	39.8
GR_30	64.4	107.2	**-28.4**	163.0.	**-3.9**	179.4	43.7
GR_25	64.2	107.8	**-28.3**	162.6	**-4.2**	179.9	43.6
GR_20	64.2	108.6	**-26.6**	162.0	**-3.0**	179.8	43.1
GR_15	64.2	108.6	**-25.7**	162.1	**-3.3**	180.8	42.6
GR_10	64.3	106.6	**-39.5**	161.2	**-2.8**	180.1	42.8
UV_30	63.6	109.0	**-27.8**	162.3	**-3.0**	181.0	44.5
UV_25	63.7	108.9	**-29.1**	162.3	**-3.5**	180.9	43.5
UV_20	63.7	109.1	**-22.7**	162.2	**-2.6**	180.3	42.9
UV_15	64.0	110.2	**-21.6**	161.6	**-2.0**	181.3	42.3
UV_10	64.0	109.7	**-37.2**	161.5	**-2.6**	180.5	43.6

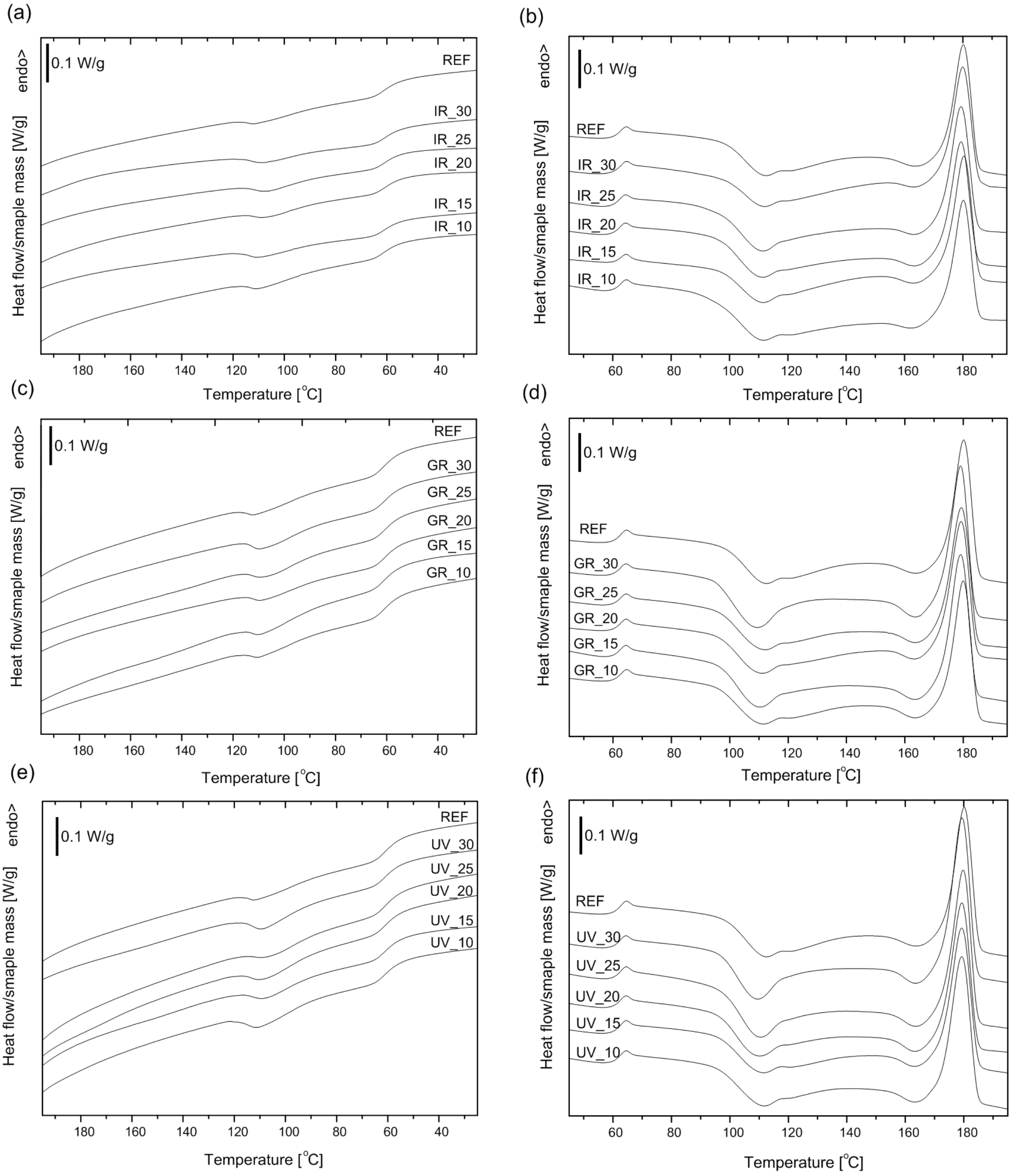

Supplementary Figure S6. The cooling (left) and 2nd heating (right) DSC curves of PLLA modified at 1030 nm (a, b), 515 nm (c, d) and 343 nm (e, f) wavelengths.

Supplementary Table S2. The thermal parameters of reference and 1030 nm, 515 nm and 343 nm laser modified PLLA samples estimated from the cooling DSC curves.

sample	T_g [^oC]	T_{cc} [^oC]	ΔH_{cc} [J/g]
REF	61.4	110.6	-2.94
IR_30	61.2	107.3	-3.53
IR_25	61.4	106.1	-3.56
IR_20	61.1	106.6	-2.92
IR_15	61.1	109.6	-3.50
IR_10	61.0	109.5	-3.51
GR_30	61.0	108.6	-4.09
GR_25	61.0	106.2	-3.53
GR_20	60.5	108.0	-3.03
GR_15	61.7	108.8	-3.45
GR_10	61.5	109.3	-2.78
UV_30	60.4	108.5	-4.86
UV_25	60.5	105.2	-3.52
UV_20	60.6	107.6	-4.52
UV_15	60.7	107.5	-3.72
UV_10	61.0	110.0	-5.24

Supplementary Table S3. The thermal parameters of reference and 1030 nm, 515 nm and 343 nm laser modified PLLA samples estimated from the second heating DSC curves.

sample	Tg [^oC]	T_{cc} [^oC]	ΔH_{cc} [J/g]	$T_{\alpha'-\alpha}$ [^oC]	T_m [^oC]	ΔH_m [J/g]
REF	62.8	112.1	-32.90	162.5	180.2	40.40
IR_30	63.0	111.8	-40.08	163.0	179.9	40.51
IR_25	62.6	111.1	-39.70	162.8	179.3	42.06
IR_20	62.6	111.0	-39.44	163.3	179.4	39.86
IR_15	62.5	111.4	-37.33	162.7	180.3	41.37
IR_10	62.9	111.3	-33.99	162.5	180.2	36.67
GR_30	62.7	109.0	-21.84	163.1	179.1	42.63
GR_25	62.8	111.6	-35.00	163.0	179.3	42.35
GR_20	62.8	110.6	-37.60	163.0	179.2	41.64
GR_15	62.7	109.8	-33.40	163.1	179.1	41.87
GR_10	63.0	111.0	-34.39	162.8	179.9	40.59
UV_30	62.8	109.1	-25.10	162.7	179.4	44.99
UV_25	62.7	110.1	-31.07	163.1	179.8	43.33
UV_20	62.8	109.6	-32.57	163.1	179.3	42.25
UV_15	62.7	111.1	-32.38	162.8	179.3	42.51
UV_10	62.7	111.1	-30.10	162.5	179.3	43.89

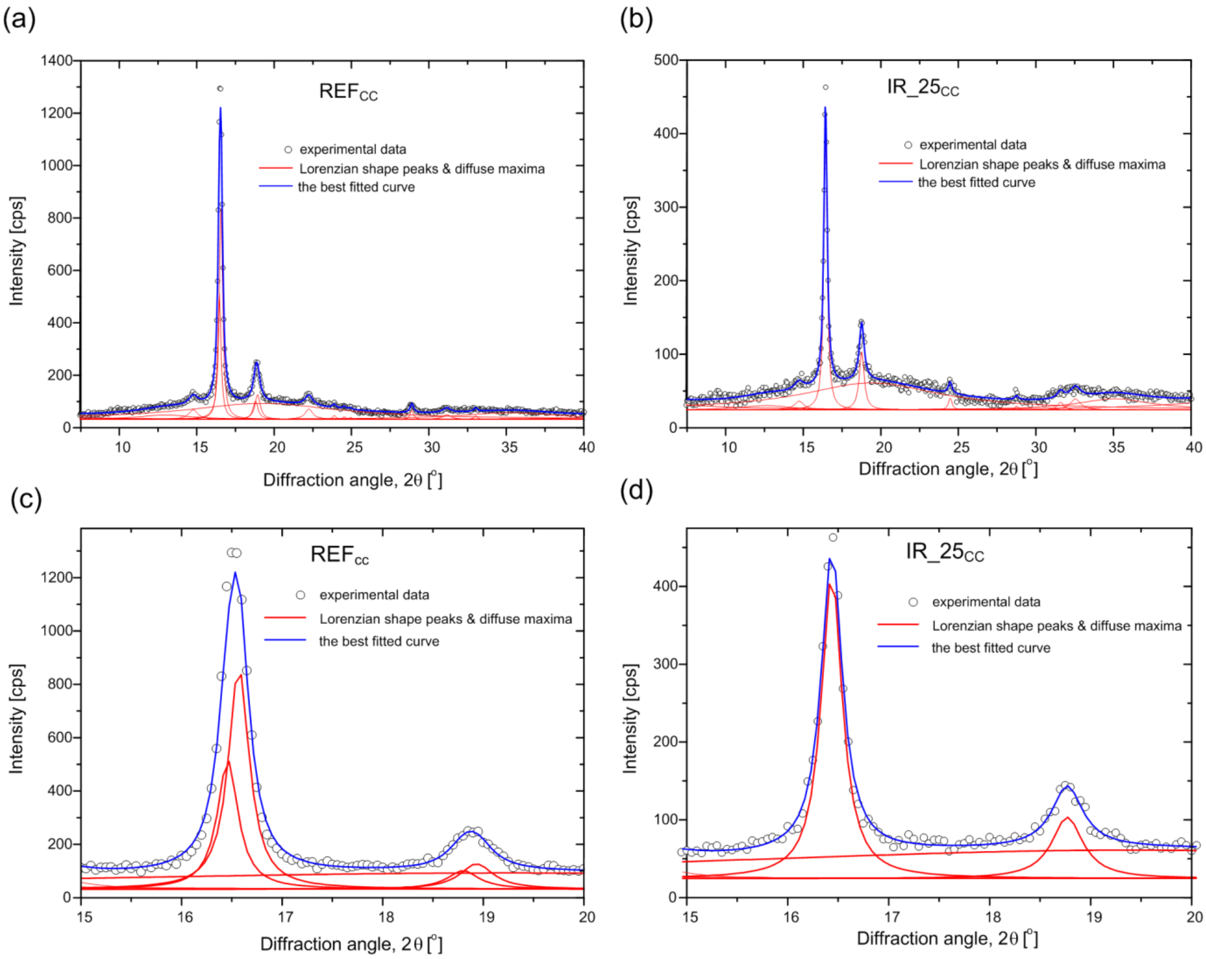

Supplementary Figure S7. Deconvolution of WAXD patterns into the diffraction peaks and diffusion maxima for nonisothermal crystallized reference PLLA and sample with filamentary modifications IR_25: full diffraction patterns (a, b); close up of main peaks (c, d)

Supplementary Table S4. The thermal parameters of laser modified PLLA at 1030 nm after hydrolysis based on the first heating DSC scan

sample	T_g [°C]	T_{cc} [°C]	ΔH_{cc} [J/g]	$T_{\alpha'\text{-}\alpha}$ [°C]	$\Delta H_{\alpha'\text{-}\alpha}$ [J/g]	T_m [°C]	ΔH_m [J/g]
REF	67.2	106.7	-39.33	161.8	-6.16	180.1	44.27
IR_30	69.3	99.4	-28.28	161.2	-6.81	179.1	49.21
IR_25	69.0	96.7	-23.43	161.0	-6.50	179.0	50.43
IR_20	69.1	100.4	-20.77	160.6	-6.77	179.1	48.32
IR_15	69.4	106.5	-26.90	161.9	-5.37	179.9	45.80
IR_10	69.4	110.0	-24.81	162.2	-4.32	180.3	43.38

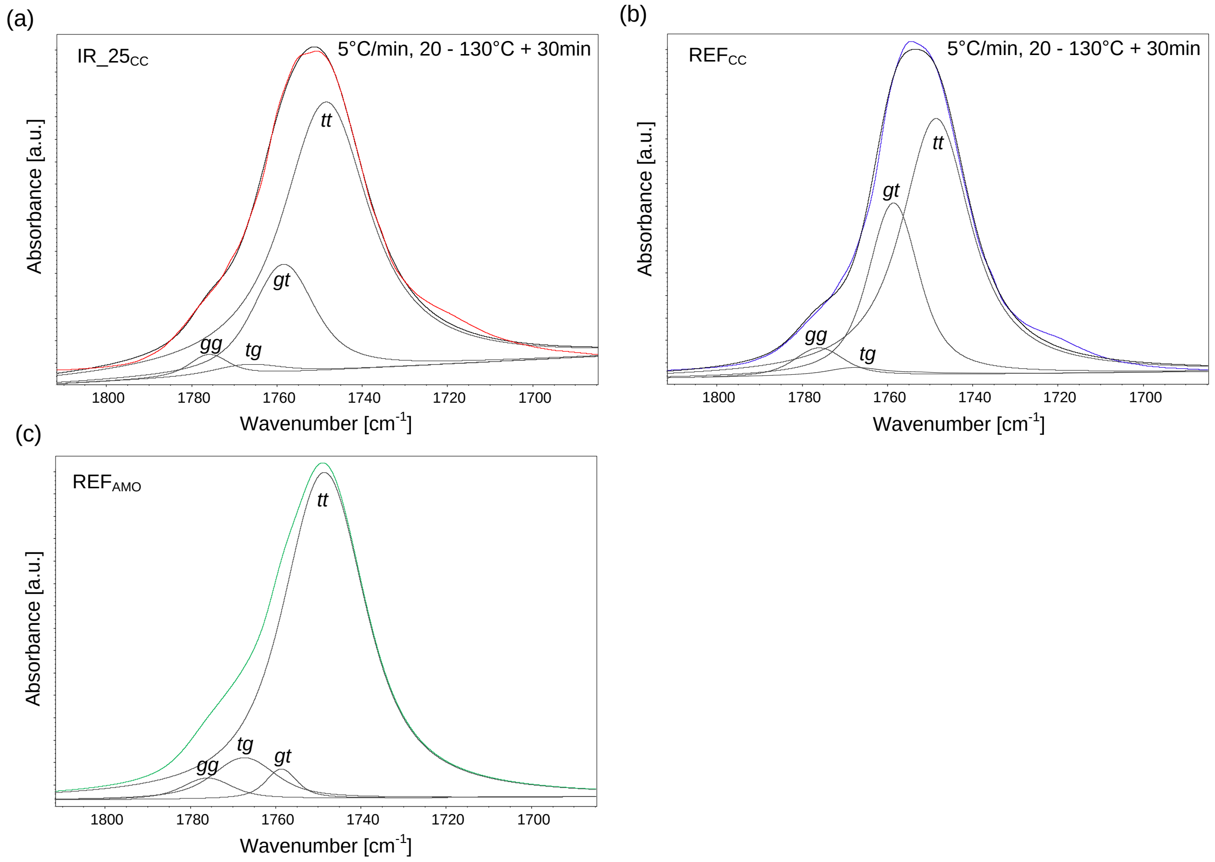

Supplementary Figure S8. Deconvolution of C=O stretching spectral region: a) cold crystallized sample with filamentary modifications IR_25; b) cold crystallized reference PLLA sample; c) amorphous reference sample. The position of peaks are following: *tt* (1749 cm^{-1}), *gt* (1759 cm^{-1}), *tg* (1767 cm^{-1}), *gg* (1776 cm^{-1})

Obtained spectra of carbonyl stretching region correlate with changes observed in 922 cm^{-1} region. The reference sample reveals greater fraction of *gt* component in relation to *tt* component what indicates higher crystalline phase perfection. Amorphous sample has mainly *tt* component and greater *tg* component.
